# Isavuconazole as initial antifungal therapy combined with surgical management of pediatric pulmonary mucormycosis: a case report and literature review

**DOI:** 10.3389/fped.2025.1701905

**Published:** 2026-01-12

**Authors:** Yao Sun, Lihua Yuan, Tianjiao Hu, Chuan Sun, Dandan Yang, Man Tian, Shichao Dong, Haiyan Gu

**Affiliations:** 1 Department of Pharmacy, Children’s Hospital of Nanjing Medical University, Nanjing, China; 2 Department of Respiratory Medicine, Children’s Hospital of Nanjing Medical University, Nanjing, China; 3 Department of Pharmacy, The Second Hospital of Tianjin Medical University, Tianjin, China

**Keywords:** diabetes, initial treatment, isavuconazole, mucormycosis, pediatric

## Abstract

Invasive mucormycosis (IM) in pediatric patients is a rare but life-threatening fungal disease with limited treatment options. Isavuconazole is a new triazole that has shown efficacy and safety in adults for both primary and salvage treatment of mucormycosis. However, data regarding the initial use of isavuconazole in children are rare. In this study, we report a case of a 6-year-old girl with diabetes mellitus. Metagenomic next-generation sequencing detected *Rhizopus oryzae* in her bronchoalveolar lavage fluid , and a chest computed tomography revealed a reversed halo sign. Oral isavuconazole was given as primary monotherapy with continuous control of blood glucose. After the lesion partially shrank and became confined, the patient visited the thoracic surgery department to undergo lobectomy; she recovered well after the procedure. This report highlights the importance of quick diagnosis of mucormycosis and may provide a reference for providing the initial antifungal treatment in pediatric mucormycosis. All of the aforementioned interventions helped buy time for subsequent surgical treatment, leading to the curing of the child. Isavuconazole may represent an effective and safe therapeutic option as first-line monotherapy for pediatric mucormycosis.

## 1 Introduction

Invasive mucormycosis (IM) is the third most common invasive fungal disease (IFD) after invasive aspergillosis (IA) and candidiasis (1). This life-threatening infection, caused by fungi of the order Mucorales, is characterized by conditional pathogenicity, aggressive tissue invasion, and high mortality. The most common pathogens are Rhizopus spp., Mucor spp., and Rhizomucor spp. (2). Epidemiological data show that pulmonary mucormycosis represents the most frequent clinical form in pediatric patients (19%–42.1%), followed by skin and soft tissue involvement (19%–21%) and rhino-orbito-cerebral disease (15.8%) (3, 4). The global SARS-CoV-2 pandemic had driven a rising incidence of mucormycosis, and diabetes mellitus is recognized as a leading risk factor, indicating the critical need for early diagnosis, timely antifungal therapy, and appropriate surgical evaluation (5). Herein, we report a pediatric case of type I diabetes mellitus complicated by pulmonary mucormycosis, successfully treated with first-line isavuconazole monotherapy combined with thoracic surgery, highlighting its potential role in pediatric antifungal stewardship programs.

## 2 Case description

A 6-year-old girl was admitted to hospital on 4 July 2023, with a 12-day history of persistent cough and intermittent fever (Tmax 39.0°C), temporarily relieved by oral ibuprofen. She experienced 4–5 daily episodes of paroxysmal cough with sputum retention. Initial chest X-ray at a referring hospital showed right upper lobe consolidation with ill-defined margins. Serology indicated mycoplasma pneumoniae infection (IgM titer 1:320) and concurrent parainfluenza virus IgM positivity. Urinalysis showed glycosuria (2+) and ketonuria (3+). The patient received intravenous azithromycin for 3 days. A computed tomography (CT) scan on June 30 revealed right upper lobe consolidation and bronchial obstruction, prompting a switch to oral erythromycin plus intravenous piperacillin–tazobactam. However, even after 48 h, she did not show improvement, following which she was hospitalized. The patient had a prior diagnosis of type I diabetes mellitus, managed with preprandial insulin (3.5 IU at breakfast and lunch, 4 IU at dinner) and basal insulin glargine (3.5 IU at bedtime). Recent glucose levels indicated poor control (fasting plasma glucose 15–20 mmol/L). On admission, her vital signs were normal. Laboratory test results revealed white blood cell count (WBC) 17.98 × 10^9^ /L, neutrophils 75.5%, lymphocytes 15.0%, C-reactive protein (CRP) 71.93 mg/L, and PCT 1.59 ng/mL. Diagnoses on admission were mycoplasma pneumoniae pneumonia and type I diabetes mellitus.

**Figure 1:**
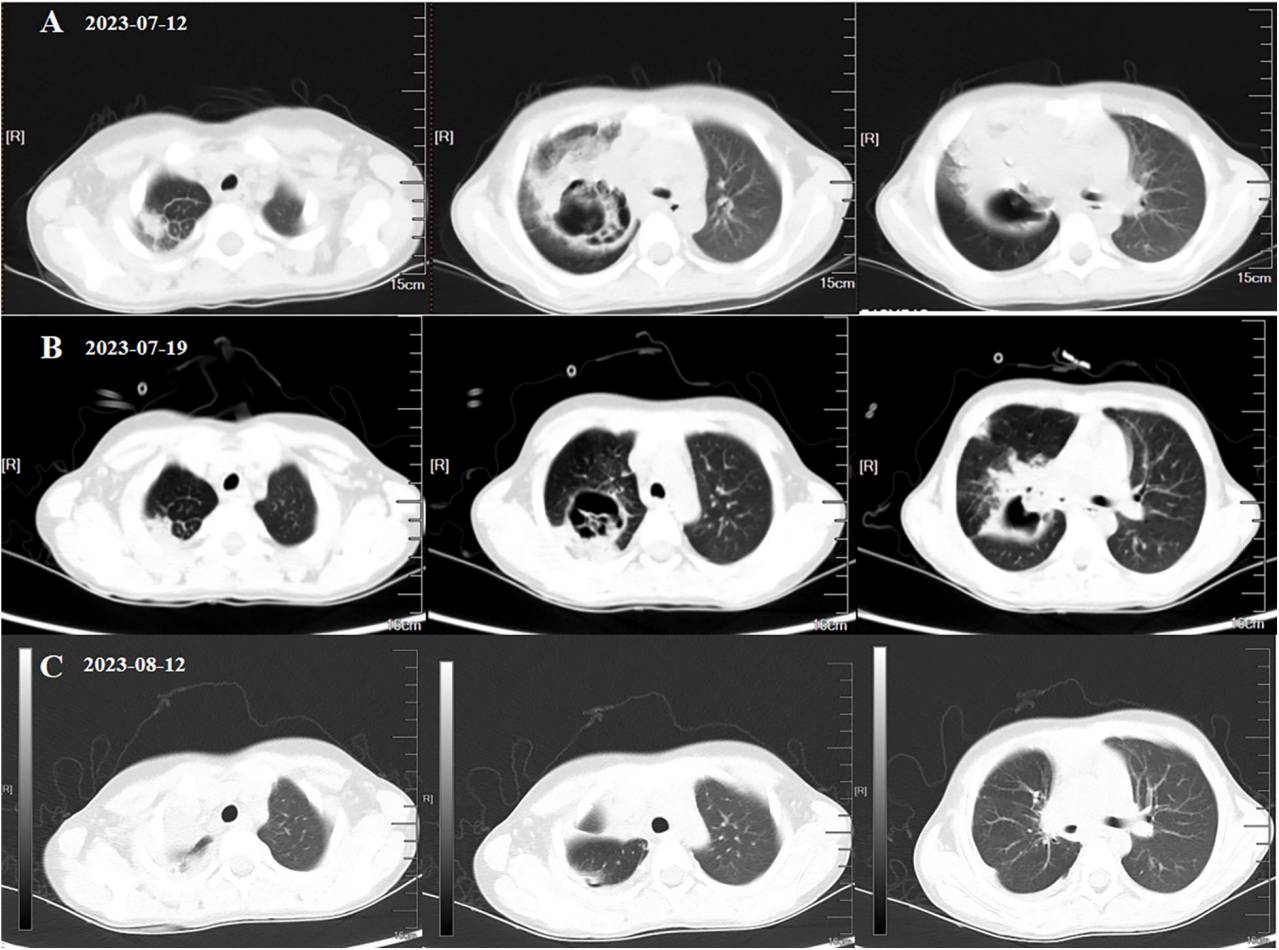
Chest CT imagings at different timelines of antibiotic therapy and surgery. **(A)** A chest CT scan taken before isavuconazole therapy. **(B)** A chest CT scan following 7 days of isavuconazole therapy. **(C)** A chest CT scan obtained 9 days postoperatively and following 28 days of isavuconazole therapy.

**Figure 2:**
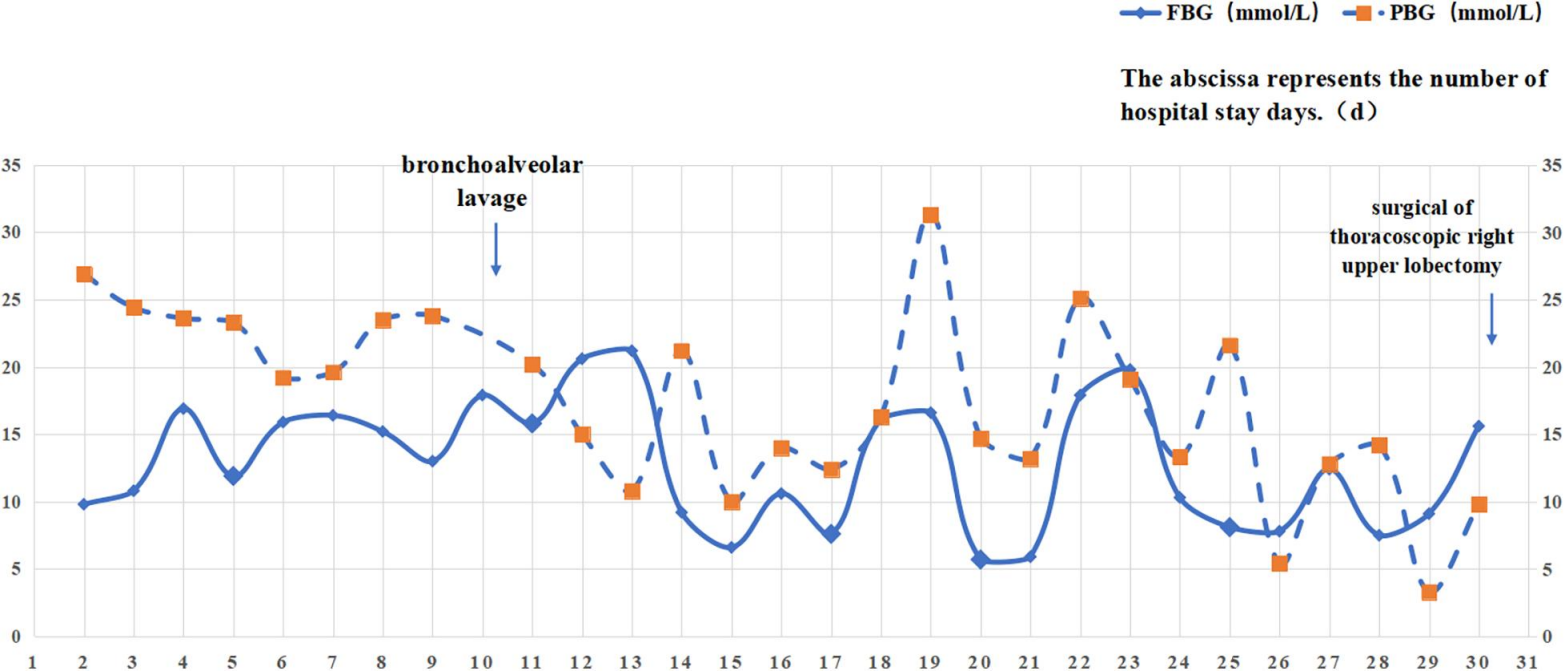
Blood glucose results (FBG and PBG) following admission. The abscissa represents the number of hospital stay days (d).

**Figure 3:**
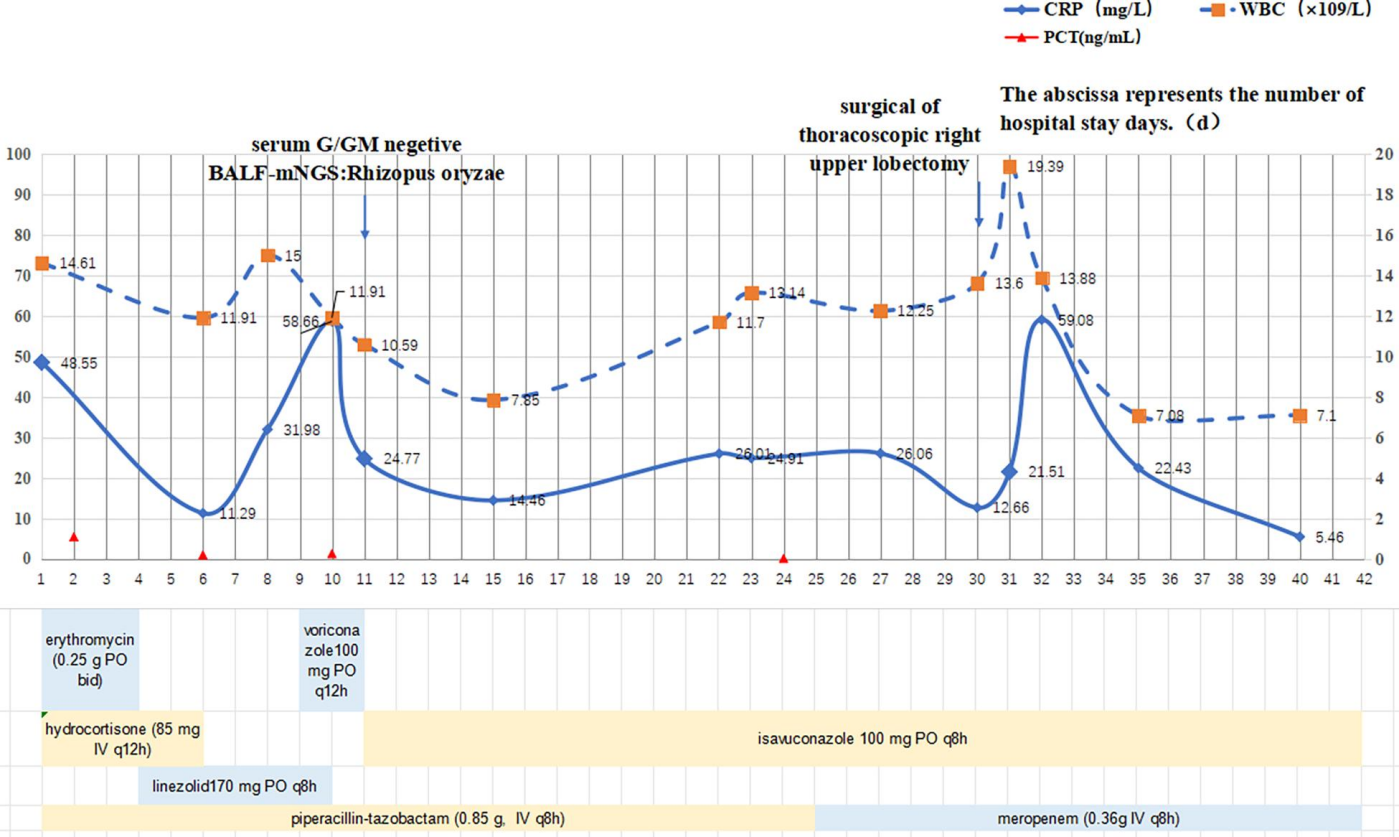
Evolution of inflammatory markers (WBC, CRP, and PCT) over time (days), with the course of antimicrobial therapy during hospitalization given below.

The patient was admitted on 4 July (day 1) and started on piperacillin–tazobactam (0.85 g IV q8h) plus erythromycin (0.25 g PO bid) and hydrocortisone (85 mg IV q12h). Despite a 4-year treatment of diabetes, she presented with severe hyperglycemia (fasting glucose 26 mmol/L), prompting insulin intensification: preprandial doses were set at 4.5, 4, and 4 IU, supplemented by an intravenous insulin micropump at 1.7 IU/h, along with dietary adjustment. On Day 4 (7 July), persistent fever (Tmax 38.2°C) led to the addition of linezolid 170 mg PO q8h for gram-positive coverage. Repeated mycoplasma pneumoniae serology and PCR test results were negative, and erythromycin was stopped because of gastrointestinal intolerance. On day 6 (9 July), persistent hyperglycemia (fasting glucose 15.9 mmol/L) required further increases in preprandial insulin to 6.5, 6, and 6 IU, while the process of insulin infusion was continued at 1.7 IU/h. Glucocorticoids were discontinued to reduce iatrogenic hyperglycemia. On day 9 (12 July), fever persisted (Tmax 38.8°C) despite negative blood cultures. Laboratory test results showed ongoing inflammation (CRP 31.93 mg/L, WBC 15.0 × 10^9^ /L, neutrophils 73.0%). A repeat chest CT revealed a reversed halo sign in the right upper lobe suggesting necrotizing pneumonia, consolidation in the right middle lobe, and air-trapping in the right lower lobe, accompanied by right pleural effusion and bronchial occlusion (Figure 1A). Given the persistent hyperglycemia (glycosuria 4+), fungal infection was strongly suspected, prompting empirical voriconazole (100 mg PO q12h).On 13 July, bronchoscopy with bronchoalveolar lavage (BAL) was performed, and in the meantime, alveolar lavage fluid was sent for metagenomic next-generation sequencing (mNGS) and serum was sent for a fungal biomarker analysis [galactomannan (GM) and 1,3-β-D-glucan (G) assays] On day 11 (14 July), GM/G test results were negative, but bronchoalveolar lavage fluid (BALF)-mNGS identified *Rhizopus oryzae* (29 unique reads) and *Hemophilus parainfluenzae* (27 unique reads). Pulmonary mucormycosis was confirmed, requiring discontinuation of linezolid and voriconazole in favor of liposomal amphotericin B (L-AmB). However, L-AmB administration requires 5% dextrose infusion, which posed significant risks to our patient given her uncontrolled diabetes (random glucose 22.1 mmol/L, ketonuria 3+; Figure 2). A multidisciplinary team recommended immediate transition to isavuconazole monotherapy with a loading dose (100 mg PO q8h × 6), followed by 100 mg PO q24h. On day 16 (19 July), a follow-up chest CT revealed consolidative changes compared with prior imaging (Figure 1B), although pleural effusion and bronchial patency alleviated. One week later (26 July), laboratory test results indicated persistent inflammation (CRP 26.01 mg/L, WBC 11.70 × 10^9^ /L, neutrophils 67.4%; Figure 3) and the patient still experienced persistent pyrexia (Tmax 38.4℃). Despite multiple insulin regimen adjustments, suboptimal glycemic control persisted. Therefore, mNGS of deep respiratory secretions was performed, revealing Klebsiella pneumoniae (540 reads) on day 25 (28 July). Piperacillin–tazobactam was replaced with meropenem (0.36 g IV q8h), while isavuconazole was continued. On day 30 (3 August), the patient was transferred to the surgical department for undergoing a thoracoscopic right upper lobectomy. On day 39 (12 August), clinical resolution was evidenced by normalized inflammatory markers and radiological clearance on CT (Figure 1C). Antifungal and antibacterial therapies were discontinued on 16 August and the patient was discharged with a scheduled outpatient review. At the 9-month follow-up (9 May 2024), port sites were well healed and a chest CT confirmed no recurrence, indicating successful treatment.

## 3 Research methods

**Figure 4:**
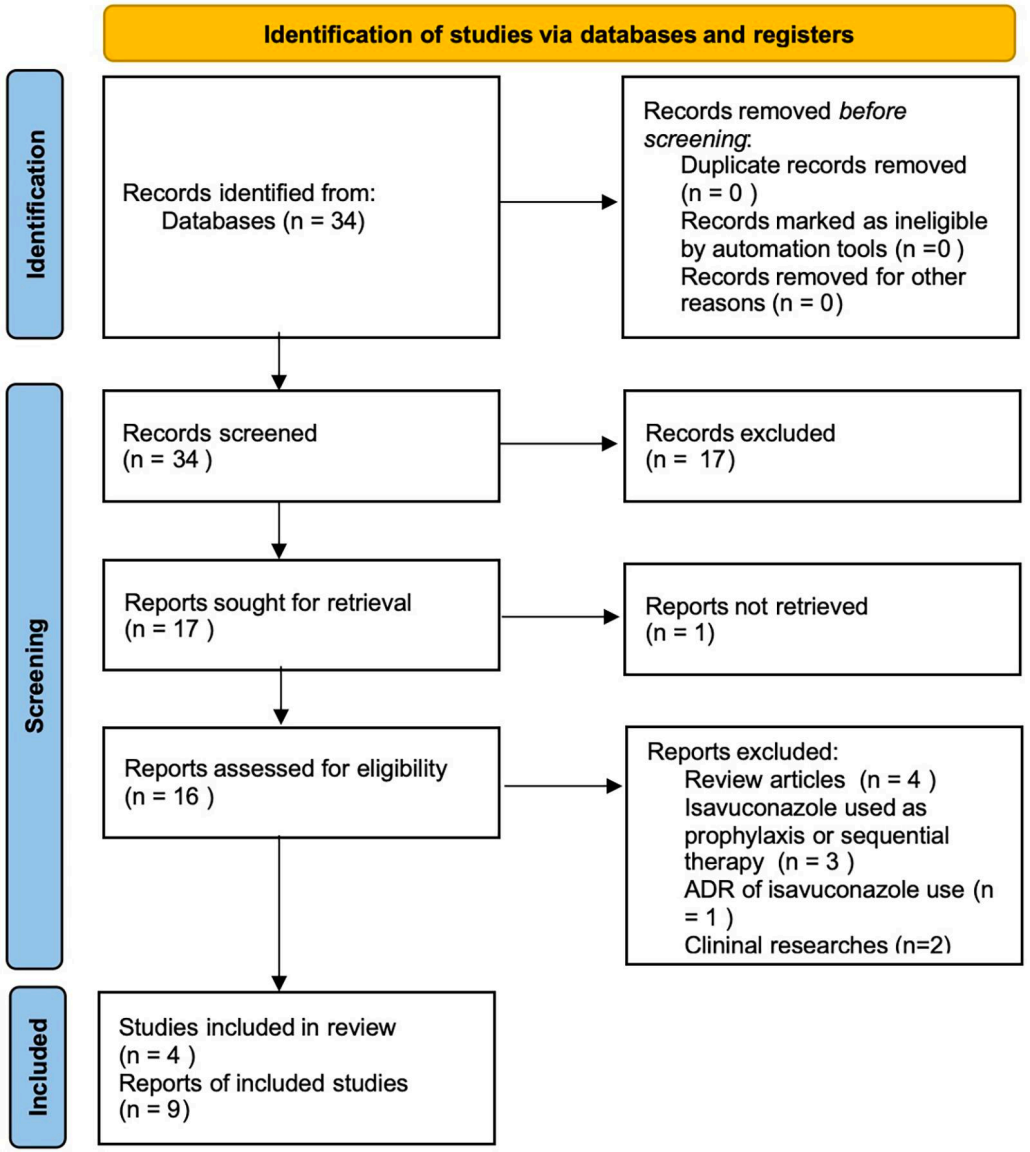
PRISMA flow diagram.

We carried out a systematic literature search in PubMed and reported the use of isavuconazole as the initial therapy of invasive mucormycosis in pediatric patients with search terms in full text: [(Mucormycosis) OR (Zygomycosis) OR (Mucormycose)] AND [(pediatric) OR (children)] AND (isavuconazole). Only case reports were included. In total, 34 articles were found. Two authors (YLH and THJ) independently collected these articles and evaluated their appropriateness based on the title and abstract. Finally, six articles including nine patients were selected. The details are provided in Table 2 and Figure 4.

## 4 Discussion

Invasive mucormycosis carries high mortality in immunocompromised adults, necessitating early diagnosis and urgent treatment; however, pediatric data on this fungal infection remain limited. Risk factors for IM include neutropenia (61.5%), broad-spectrum antibiotic use (35%), and diabetes mellitus (27%), according to a retrospective study based on PubMed and Cochrane databases (6). Diabetes was found to be the most common risk factor (37.2%) in a Chinese retrospective study (4). In this case report, we describe the successful diagnosis of *Rhizopus oryzae* in a child with diabetes confirmed by mNGS and the treatment of isavuconazole as initial therapy, enabling subsequent surgical intervention.

The ESCMID/ECMM guidelines strongly recommend high-dose liposomal amphotericin B (L-AmB) as first-line therapy, with moderate support for isavuconazole or posaconazole, while advising against amphotericin B deoxycholate because of toxicity (7). International registry data confirm that combining systemic antifungals with surgery improves survival rates (3). Although L-AmB offers better tolerability than conventional formulations, high-dose regimens (5–10 mg/kg/day) carry nephrotoxicity risks. A retrospective study of 119 patients with mucormycosis treated with L-AmB showed that 16 (13.4%) discontinued the drug because of adverse effects, mainly caused by renal dysfunction (21/41) (4). Newer triazoles such as posaconazole and isavuconazole, with fewer adverse effects, are emerging as alternatives. Isavuconazole, a broad-spectrum triazole, was approved in the United States in March 2015 for the treatment of IA or IM in adults, as well as in Europe in October 2015 for the treatment of adults with IA or adults with IM for whom amphotericin B is inappropriate. However, pediatric experience remains limited, with few reports on its safety and efficacy in children (Table 1). At the time of treatment, isavuconazole was not yet approved for pediatric use; FDA approval for children aged ≥6 years (weight ≥16 kg) was granted in December 2023. Informed consent was obtained from the parents of the child. Malkiel described three pediatric hematology-oncology patients (aged 4.5–19 years) with progressive mucormycosis despite surgery and amphotericin B, who improved after switching to isavuconazole without adverse events (8). Pomorska reported a 7-year-old girl with ALL and pulmonary mucormycosis who received surgical debridement and combination antifungals including isavuconazole for 8 months, achieving complete radiographic resolution (6). These reports suggest that isavuconazole may be safe and effective in children. Supporting this, the adult VITAL trial found isavuconazole non-inferior to amphotericin B for initial or salvage therapy of IM, with comparable response and mortality rates (9).

**Table 1:** Comparison between liposomal amphotericin B (L-AmB) and isavuconazole.

Factor	Liposomal amphotericin B (L-AmB)	Isavuconazole
Efficacy	Highly effective for severe systemic fungal infections (e.g., cryptococcosis, aspergillosis, and mucormycosis)	Effective against a broad range of fungi, particularly Aspergillus and Mucorales. Less effective for Cryptococcus compared with L-AmB.
Safety	Generally safe, but can cause nephrotoxicity, infusion-related reactions, and electrolyte disturbances. Requires monitoring of renal function.	Has better renal safety profile than L-AmB, but still potential for hepatotoxicity, QT interval prolongation, and drug–drug interactions (especially with CYP enzymes).
Tolerability	Well-tolerated with less risk of nephrotoxicity compared with conventional amphotericin B. Infusion-related reactions (fever, chills) can occur.	Generally well-tolerated. Common side effects include GI disturbances (nausea, diarrhea), headache, and elevated liver enzymes.
Pediatric-specific considerations	Approved for pediatric use (including neonates) with careful monitoring of renal function and electrolytes. Dosing is based on body weight.	Approved for pediatric use (aged 2 years and older). Dosing is based on age and weight, with caution recommended for patients with hepatic impairment.

Only six reports have described isavuconazole as first-line therapy for pediatric invasive mucormycosis, however with inconsistent outcomes (Table 2). Of these, three employed isavuconazole alone initially and sequentially (10–12), one used it as initial treatment followed by sequential treatment with L-AmB (13), and two combined L-AmB with isavuconazole from the start of treatment (5, 14). Among the total nine children, three were cured, two died, and outcomes for the remainder are unknown. Zhang et al. reported that a 14-year-old boy without underlying disease was diagnosed with *Rhizopus oryzae* by BALF-mNGS and cured with isavuconazole monotherapy (10). Cornu and Bruno described the case of a 3-year-old girl with ALL and disseminated mucormycosis, who responded to isavuconazole alone, and then this was combined with L-AmB (13). Sosnowska-Sienkiewicz presented a complicated management in a 16-month-old child with leukemia and generalized mucormycosis localized in the liver and gastrointestinal tract (5). A 6-month course of isavuconazole and L-AmB as well as surgical treatment led to the cure of infection. Ferdjallah reported an 8-year-old boy with adrenoleukodystrophy who was diagnosed with rhinocerebral mucormycosis (RCM) and pulmonary mucormycosis after allogeneic hematopoietic cell transplantation (allo-HCT) (14). He was treated with isavuconazole in combination with L-AmB, but he died on day 22 post transplant. In the same year, Mahan reported a 16-year-old boy with RCM who had both diabetic ketoacidosis (DKA) and internal carotid artery occlusion (11). After being diagnosed, he received treatment with isavuconazole alone, but he died of cerebral edema and herniation at a later stage of the disease. In Decembrino's report, it was stated that ISA was used as first-line monotherapy in four children with hemato-oncological diseases; however, specific clinical outcomes remained unknown (12).

**Table 2:** Summary of literatures reported the use of isavuconazole as the initial therapy of invasive mucormycosis in pediatric patients.

Case report/series	Country	Disease	Age	Gender	Comorbidities	Loading dose of ISA	Maintenance dose of ISA	Duration of treatment	Other treatments followed	Outcome
Cornu (13)	France	Disseminated mucormycosis	3 years	F	ALL	70 mg q8h for 48 h ivgtt	70 mg QD	24 m	Then LAMB	cured
Decembrino (12)	Italy	NA	NA	NA	NA	ISA was used as first-line therapy, in four patients	NA	NA	NA	NA
Mahan (11)	America	RCM	16 years	M	DKA/Internal Carotid Artery Occlusion	NA	NA	NA	None	Death
Ferdjallah (14)	America	RCM	8 years	M	adrenoleukodystrophy	7 mg/kg q8h for 48 h ivgtt	7 mg/kg QD	NA	/+LAMB	Death
Sosnowska-Sienkiewicz (5)	Norway	mucormycosis localized in the liver and gastrointestinal tract	16 months	F	ALL	10 mg/kg q8h for 48 h ivgtt	10 mg/kg QD	NA	/+LAMB	Cured
Zhang (10)	China	Pulmonary	14	M	None	NA	NA	NA	None	Cured

ISA, isavuconazole; RCM, rhinocerebral mucormycosis; DKA, diabetic ketoacidosis; LAMB, liposomal amphotericin; ALL, acute lymphoblastic leukemia.

These reports provide a new perspective on isavuconazole as a potential alternative to liposomal amphotericin B (L-AmB) for pediatric mucormycosis. Despite L-AmB being the guideline-recommended first-line treatment, it was unavailable at our center, and therefore, only amphotericin B deoxycholate (AmBd) could be offered to our patient. However, AmBd must also be prepared in 5% dextrose solution, which again posed a substantial risk to this patient who had poorly controlled diabetes, as dextrose infusion would likely exacerbate glycemic instability. Studies have shown that hyperglycemia may increase the risk of mucormycosis through mechanisms such as causing a decrease in phagocytic activity, an increase in free iron concentration, and an upregulation of the expression of glucose-regulated protein (GRP-78) (15). After weighing these factors, our team decided to use isavuconazole as an alternative antifungal regimen.

Since isavuconazole was not approved for children at the time, there was no standard pediatric dose. Ashkenazi-Hoffnung successfully treated four children (7–14 years’ old) at two pediatric tertiary centers between 2015 and 2019, using a median dose of 5.0 mg/kg/day (range 3.5–5.6 mg/kg/day) for 2–4 months (16). Malkiel described the cases of two younger children (4–4.5 years) initially dosed at 4.2–5.6 mg/kg/day and later switched to the adult fixed dose (200 mg q24h) because of subtherapeutic levels (8). Marjorie used a reduced dose (70 mg IV q8h, then 70 mg daily) in a 3-year-old child with renal impairment, achieving remission after 150 days (13). Pharmacokinetic studies suggest that children may require higher doses because of their enhanced metabolism and clearance (12). Phase 1 data indicate the lowest exposure in adolescents (12-18 years’ old), while recent work notes that children aged 1–3 years often fail to reach target levels at studied doses and may need adult-equivalent dosing (17, 18). Since the absolute rate of bioavailability of oral isavuconazole was reported to be 98% (19), we decided to give isavuconazole capsules to our patient for oral treatment. Our patient (17 kg, normal renal function) received a loading dose of 100 mg q8h for 48 h, followed by 100 mg daily. Within 10 days, her fever resolved, cough subsided, and CT findings stabilized, which made it possible to proceed with the surgical intervention.

Early recognition of mucormycosis is challenging due to its non-specific symptoms and signs. Current diagnosis relies primarily on imaging, histopathology, and mycological culture (20). Conventional culture is hindered by the fastidious nutrition and slow growth of Mucorales, yielding very low positivity rates. Molecular assays lack sensitivity and breadth, and mNGS delivers high sensitivity rates, rapid turnaround, and rare-pathogen coverage, markedly lifting clinical detection rates. It provides key evidence for early targeted antifungal therapy and prognostic assessment, which raises survival rates from 33% to 61% (21). In the early stages of treatment in our patient, the patient showed poor response to conventional anti-infective therapy, and chest CT imaging revealed the characteristic “reversed halo sign.” Combined with her medical history and clinical symptoms, we strongly suspected mucormycosis. Consequently, bronchoscopy was immediately performed, and BALF-mNGS identified *Rhizopus oryzae*. The timely pathogenic detection enabled us to establish a diagnosis and initiate early intervention, which laid the foundation for her subsequent treatment—the combined regimen of targeted antifungal therapy, antibacterial coverage, and surgical resection. Isavuconazole provided reliable antifungal activity against *Rhizopus oryzae*, while meropenem was essential for coinfection management. Surgical debridement then removed necrotic tissues that are characteristically impermeable to systemic antifungals, thereby eliminating the primary nidus for recurrence. This integrated strategy demonstrated synergistic efficacy in achieving disease control and improving chances of survival.

Current guidelines are inconclusive about the need for therapeutic drug monitoring (TDM) with isavuconazole. Kaindl et al. demonstrated that over 90% of patients receiving standard-dose isavuconazole achieved trough concentrations exceeding the therapeutic threshold (>1 mg/L) (22). However, TDM may be warranted in specific clinical scenarios, such as morbidly obese patients or those receiving concomitant medications with drug–drug interactions, based on individualized clinical judgment (23). Compared with other triazole antifungals, isavuconazole exhibits favorable safety and tolerability profiles. The most common adverse events include gastrointestinal symptoms (nausea, vomiting, diarrhea) and transient hepatic function abnormalities, typically not requiring treatment discontinuation. In the SECURE trial, it was found that the incidence of drug-related adverse events was significantly lower with isavuconazole than with voriconazole (42.4% vs. 59.8%; *P* < 0.001) (24). In the case of our patient, the child was not prescribed concomitant medications with isavuconazole-related drug–drug interactions. The combination of pharmacological therapy and surgical intervention demonstrated clinical efficacy, with periodic therapeutic assessments and close monitoring of hepatic/renal function, inflammatory markers, and gastrointestinal status. No drug-related toxicities were observed during treatment. However, whether TDM should be routinely implemented requires further investigation.

The patient received follow-up care in the Department of Respiratory Medicine for nine months postoperatively, after which long-term monitoring was transitioned to the endocrinology outpatient clinic because of her pre-existing diabetes. Inflammatory markers were assessed at 1 week and 1 month after surgery, with both results showing abnormalities. As the child remained asymptomatic, further routine monitoring of inflammatory markers was deemed unnecessary and was therefore discontinued. Serial chest CT scans were performed at 1 week and 1, 2, 6, and 9 months after surgery to evaluate lesion resolution. The patient demonstrated satisfactory postoperative recovery; however, diabetes remains a persistent risk factor for mucormycosis recurrence. In collaboration with endocrinology specialists, the patient's long-term follow-up is being managed through the endocrinology clinic, with particular attention to glycemic control. We have recommended annual chest CT surveillance in this patient in the event of suboptimal diabetes management. However, for patients receiving prolonged isavuconazole, liver function tests and electrolyte panels should be performed on a monthly basis.

## 5 Conclusion

Invasive mucormycosis initially presents with non-specific fever and cough. Early diagnosis based on clinical manifestations and imaging findings is critical. As an adjunct diagnostic tool, mNGS shows superior sensitivity and shorter turnaround time compared with conventional culture-based methods or histopathology. In the case of the patient in this study, the characteristic “reversed halo sign” on chest CT combined with BALF-mNGS identification of *Rhizopus oryzae* confirmed the diagnosis. Off-label isavuconazole, chosen for reasons of availability and patient status, halted progression of the infection and enabled deferred surgery; no adverse events occurred in the patient, and 4-week imaging showed complete resolution. This case represents a rare report of pulmonary mucormycosis secondary to diabetes mellitus treated with isavuconazole as first-line monotherapy. Since our experience is based on a single case, which suggests that isavuconazole may be a potent therapeutic option when L-AmB is unavailable, further validation through additional clinical cases is required. Given the limited pediatric data, our experience provides valuable evidence for antifungal selection in childhood mucormycosis.

## Data Availability

The original contributions presented in the study are included in the article/Supplementary Material, and further inquiries can be directed to the corresponding author.
